# Climate change and the emergence of vector-borne diseases in Europe: case study of dengue fever

**DOI:** 10.1186/1471-2458-14-781

**Published:** 2014-08-22

**Authors:** Maha Bouzid, Felipe J Colón-González, Tobias Lung, Iain R Lake, Paul R Hunter

**Affiliations:** Norwich Medical School, University of East Anglia, Norwich, UK; School of Environmental Sciences, University of East Anglia, Norwich, UK; The Abdus Salam International Centre for Theoretical Physics, Earth System Physics Section, Trieste, Italy; Joint Research Centre, European Commission, Institute for Environment and Sustainability, Ispra, Italy; European Environment Agency, Copenhagen, Denmark

**Keywords:** Dengue fever, Climate change, Europe, Disease risk

## Abstract

**Background:**

Dengue fever is the most prevalent mosquito-borne viral disease worldwide. Dengue transmission is critically dependent on climatic factors and there is much concern as to whether climate change would spread the disease to areas currently unaffected. The occurrence of autochthonous infections in Croatia and France in 2010 has raised concerns about a potential re-emergence of dengue in Europe. The objective of this study is to estimate dengue risk in Europe under climate change scenarios.

**Methods:**

We used a Generalized Additive Model (GAM) to estimate dengue fever risk as a function of climatic variables (maximum temperature, minimum temperature, precipitation, humidity) and socioeconomic factors (population density, urbanisation, GDP per capita and population size), under contemporary conditions (1985–2007) in Mexico. We then used our model estimates to project dengue incidence under baseline conditions (1961–1990) and three climate change scenarios: short-term 2011–2040, medium-term 2041–2070 and long-term 2071–2100 across Europe. The model was used to calculate average number of yearly dengue cases at a spatial resolution of 10 × 10 km grid covering all land surface of the currently 27 EU member states. To our knowledge, this is the first attempt to model dengue fever risk in Europe in terms of disease occurrence rather than mosquito presence.

**Results:**

The results were presented using Geographical Information System (GIS) and allowed identification of areas at high risk. Dengue fever hot spots were clustered around the coastal areas of the Mediterranean and Adriatic seas and the Po Valley in northern Italy.

**Conclusions:**

This risk assessment study is likely to be a valuable tool assisting effective and targeted adaptation responses to reduce the likely increased burden of dengue fever in a warmer world.

**Electronic supplementary material:**

The online version of this article (doi:10.1186/1471-2458-14-781) contains supplementary material, which is available to authorized users.

## Background

Several vector-borne diseases are spread in Europe and the effect of climate change on disease distribution has been extensively discussed [1–5]. Most authors consider that climate change is likely to have greatest impact on dengue fever, West Nile fever, chikungunya fever, malaria, leishmaniasis, tick-borne encephalitis, Lyme borreliosis, Crimean-Congo haemorrhagic fever, spotted fever rickettsioses, Yellow fever and Rift Valley fever. One disease that has received much interest in recent years is dengue fever. Dengue is a mosquito-borne disease caused by an RNA virus of the genus *Flavivirus*. Uncomplicated dengue can present with fever, headache and muscle and joint pains. A proportion of infections can develop into severe forms namely dengue haemorrhagic fever and dengue shock syndrome, which are associated with higher mortality rates. Dengue fever is endemic in over 100 countries in Africa, the Americas, the Eastern Mediterranean, South-east Asia and the Western Pacific, with the last two regions being the most seriously affected [6]. It is estimated that over 50 million new dengue fever infections and approximately 12,000 deaths, mainly among children, occur worldwide every year [7].

There has been a significant global increase in dengue incidence and it is currently considered the most important human arboviral disease worldwide. The successful spread of dengue has been attributed to various factors including population growth, urbanization, global travel, and environmental conditions. In Europe, dengue fever is rare but cases are imported every year by tourists returning from endemic areas. Recently, autochthonous dengue cases have been reported in Croatia and France, highlighting the suitability of these regions for dengue transmission [8, 9]. These cases have raised concerns about the potential for the emergence of dengue fever in Europe especially with predicted climate change.

One of the reasons for these concerns is that dengue vectors are already present within Europe. *Aedes (Stegomyia) aegypti (Linneaus)* is the major urban vector of dengue worldwide [10]. *A. aegypti* is closely associated with humans and human habitations. Female mosquitoes lay their eggs on or near water surface in natural or artificial containers [10, 11]. *Aedes albopictus* is the secondary vector of dengue fever and is adapted to the peridomestic environment [12]. According to the “European Network for arthropod vector surveillance for human public health” (VBORNET) (http://www.vbornet.eu/, last accessed June 2014), *A. albopictus* is present in many European countries: Spain, France, Switzerland, Italy, Slovenia, Croatia, Bosnia and Herzegovina, Serbia, Montenegro, Albania, Greece, Monaco, San Marino, Bulgaria, the Netherlands and Russia. By contrast, *A. aegypti* has only been reported from Madeira, the Netherlands, Georgia and southern Russia.

There is much debate about how future climate change will affect dengue risk, especially in countries where the disease is not currently endemic [10, 13, 14]. Recent studies have modelled the future dengue distribution under predicted climate change either on a global scale [15–19] or in endemic countries [20, 21]. These models have suggested a latitudinal and altitudinal expansion of the geographical range of dengue.

In Europe, there have been too few dengue cases to conduct a rigorous analysis. Consequently, estimation of dengue risk has so far relied on past, current and projected future distribution of *A. albopictus* [22] *.* Although presence of the vector is necessary for dengue to become endemic, vector presence is not sufficient in itself to determine disease occurrence [23]. The objective of this study is to model dengue risk based on clinical data. We have used one of the largest and more spatially diverse dengue dataset yet assembled to compute significant relationships between dengue and weather parameters [24]. Subsequently, the model outputs were used to project dengue risk across Europe under climate change scenarios.

## Methods

### Mexican data

The dengue dataset was primarily developed for a study of the effects of weather on dengue incidence across Mexico [24]. Dengue data comprised state-specific monthly reports of laboratory confirmed dengue cases, retrieved from the Mexican Health Secretariat (http://www.epidemiologia.salud.gob.mx/anuario/html/anuarios.html, last accessed June 2014) for the period January 1985 to December 2007. Monthly average minimum and maximum temperatures and monthly precipitation for each state were provided by the Mexican National Meteorological Service. Monthly mean humidity was retrieved from the National Centers for Environmental Prediction and National Center for Atmospheric Research (NCEP/NCAR) “Reanalysis 1” (http://www.esrl.noaa.gov/psd/data/gridded/data.ncep.reanalysis.pressure.html, last accessed June 2014). Yearly Gross domestic product (GDP) per capita (PPP in constant 2005 international dollars) was obtained from the World Bank at the national level (http://data.worldbank.org/country/mexico, last accessed June 2014). State-specific GDP estimates were computed as previously described [24]. The proportion of people living in urban areas was retrieved from the Mexican Chamber of Deputies (http://www.cefp.gob.mx/intr/bancosdeinformacion/estatales/indicadores_socioeconomicos/is003.xls, last accessed June 2014). Population density was calculated by normalising population to state area size. Table 1 presents the summary statistics for these variables.

**Table 1:** **Summary of statistical characteristics of the climatic and socioeconomic variables used for this study in Mexico and Europe**

	Mean	s.d	Min	Max
**Mexican data**				
Population density	260.22	989.96	3.6	5923.8
Urban population	71.72	15.59	35.72	100
GDP	10.55	5.11	4.31	33.16
Tmin	13.29	5.58	-2.87	24.88
Tmax	28.5	4.39	13.32	39.95
Precipitation	72.73	88.49	0	802.45
Humidity	70.79	17.66	13.34	97.41
**European data**				
**Baseline conditions**				
Population density	105.06	360	0	14820.6
Urban population	16.82	31.3	0	100
GDP	23.57	8.78	4.62	129
Tmin	4.52	6.92	-16.57	24.01
Tmax	12.3	10.05	-11.84	44.09
Precipitation	70.98	39	0	620.19
Humidity	81.08	14.01	25.69	97.75
**2011-2040**				
Tmin	5.01	7.24	-15.79	24.75
Tmax	12.82	10.5	-10.55	44.45
Precipitation	69.6	39.53	0	650.34
Humidity	80.5	14.76	25.82	97.99
**2041-2070**				
Tmin	6.60	6.91	-14.44	26.66
Tmax	14.52	10.4	-8.88	46.46
Precipitation	69.98	39.64	0	628.87
Humidity	79.76	15.89	24.35	98.25
**2071-2100**				
Tmin	7.92	6.88	-12.21	28.4
Tmax	15.88	10.54	-7.65	48.45
Precipitation	70.28	42.21	0	718.44
Humidity	79.26	16.51	23.65	98.22

s.d: standard deviation, Min: minimum value, Max: maximum value. Tmin: minimum temperature, Tmax: maximum temperature. Units of measures: Population density (number of people/km^2^), Urban population (% population living in urban areas), GDP (thousand International dollars), Temperature (degrees Celsius), Precipitation (milimetres), Humidity (milibars).

### Model calibration

Generalized Additive Models (GAMs) are semi-parametric extensions of the generalized linear model (GLM), where the linear predictor Σ*β*_*j*_*X*_*j*_ is replaced by a sum (hence the name additive) of smooth functions of covariates Σ*s*_*j*_(*X*_*j*_) [25]. Like in GLMs, GAMs allow the exploration of nonlinear data structures in the context of exponential family distributions (e.g. Poisson and Binomial), and use link functions to establish relationships between the mean of the outcome variable and the predictors [26, 27]. Unlike GLMs, GAMs automatically identify and estimate the optimal degree of nonlinearity of the model directly from the data [28]. In our study, the expected number of dengue cases *E*(*y*_*ti*_) ≡ *μ*_it_ for State *i* at time *t* was assumed to follow an overdispersed Poisson distribution described by:


where *g*(.) is a log link function of the expectation *μ*_it_ ≡ *E*(*y*_*ti*_) with *y*_*ti*_ denoting the time series of dengue counts. The logarithm of the population (*ξ*) at time *t* and state *i* is included as an exposure variable to standardise the dengue data by population. Weather has a delayed effect on dengue incidence. Therefore, we specified our *j*-th meteorological variables *X*_*jti*_ within biologically and physically plausible time lags based on literature reports in Mexico [29–31]. Weather variables comprised average monthly minimum (Tmin) and maximum (Tmax) temperatures, monthly precipitation (Precip) and average monthly relative humidity (Humid). All weather parameters were lagged 1 and 2 months (Tmin_1:2_, Tmax_1:2_, Precip_1:2_, Humid_1:2_). The term *s*_*j*_(.) corresponds to univariate smooth functions defined by penalized cubic regression splines. We adjusted our model for the effects of socioeconomic variables *Z*_*kit*_ represented by GDP per capita, the proportion of the population living in urban settlements and population density. Socioeconomic variables entered the model linearly. Analyses were conducted in R version 2.15.0 [32].

Many epidemiological datasets are likely to be dominated by long-term and seasonal trends. Therefore, adjusting the regression models for these patterns is necessary to separate them from the effects of weather parameters on the health variable [33]. Our model does not account for seasonal trends as seasonality for Europe is unlikely to be similar to Mexico given the wider range of temperatures between summer and winter. Although mosquito presence is a key factor in the epidemiology and occurrence of the disease, to our knowledge there are no state-specific long-term time series of mosquito presence across Mexico. Consequently, data on mosquito presence could not be incorporated into our model. The GAM-estimated relationships between dengue, weather and socioeconomic development in Mexico were then used to project dengue fever risk across Europe.

### European data and dengue fever risk modelling

European climate data were retrieved from the regional climate model COSMO-CLM (CCLM), forced with output from the coupled atmosphere–ocean global climate model (GCM) ECHAM5/MPIOM [34]. These regional simulations represent aerosol and GHG forcing according to the A1B scenario of the Special Report on Emissions Scenarios (SRES) of the IPCC [35]. A1B corresponds to a projected increase in global surface temperature of 2.8°C in 2090–2099 (relative to 1980–1999) and a likely range of up to 4.4°C [36]. It assumes rapid economic growth, rapid introduction of efficient technologies, convergence among regions and a balance across energy sources. The regional climate data correspond to the period 1961–2100, with a domain covering the entire European continent at a resolution of about 18 × 18 km. Data were re-scaled to a grid cell size of 10 × 10 km for the purpose of this study. The same four monthly climatic variables (Tmin, Tmax, Precip, Humid) lagged 1 and 2 months, as used for model calibration with the Mexican data, were calculated over four time periods, (a) baseline 1961–1990, (b) short-term scenario 2011–2040, (c) medium-term scenario 2041–2070, and (d) long-term scenario 2071–2100.

GDP per capita data were retrieved from EUROSTAT (in Euros) and converted into constant 2005 international dollars to be concordant with the Mexican data used for model calibration. Country level data from the World Development Indicators dataset (http://databank.worldbank.org/data/Databases.aspx, last accessed June 2014) were disaggregated to NUTS-3 level (Nomenclature of territorial units for statistics) by using the NUTS-3 level shares for each country as calculated from EUROSTAT. Then, an areal weighting approach was employed to convert the NUTS-3 data into the 10 × 10 km grid (see Additional file 1). Areal weighting is commonly used to transform administrative boundary data to raster format, whereby each grid cell is assigned a value according to the percentage of its area covered by the overlying administrative region [37].

The proportion of population living in urban areas and total population data were retrieved from the GEOSTAT 2006 population grid dataset of the European Forum for Geostatistics (EFGS) (http://www.efgs.info/, last accessed June 2014) at a spatial resolution of 1 × 1 km. Urban clusters were defined by two criteria first each grid cell of 1 × 1 km must have a minimum population density of 300 people per km^2^ and second clusters of adjoining grid cells must accommodate at least 5000 people, in line with the definitions used by the European Commission [38]. The total number of urban population for each 10 × 10 km grid cell was extracted and divided by total population to obtain proportion of population in urban area (see Additional file 1). Due to the lack of projections both in terms of SRES scenario and spatial detail, the socioeconomic variables were held constant at their mean value for baseline conditions in order to isolate the effects of climate.

### Mapping of dengue fever risk

The model was used to project monthly dengue cases, which were aggregated to calculate the average number of cases per year for each time period. These were used to generate dengue risk maps using ArcGIS 10.1 (http://www.esri.com/software/arcgis, last accessed June 2014). In total, four maps were produced corresponding to the time periods of study. Identical class sizes were applied across all four time periods in order to ensure that value changes could be observed over time. The map series employ a bipolar hue progression [39] ranging from green (no/low risk areas) to bright red (areas with the highest dengue risk). Moreover, a second map series was generated, that normalises dengue cases by total population to derive dengue incidence. We used colours ranging from blue for no/low risk to cherry brown for high risk areas. In addition, standard error for each grid cell was calculated. Standard error values were subjected to the same aggregation and averaging procedure as for dengue number of cases and dengue incidence and were used to produce maps of uncertainty.

## Results

**Figure 1:**
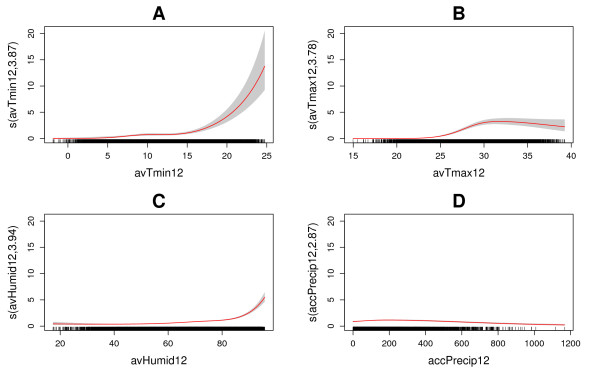
**GAM-estimated relationships between average monthly dengue cases and average monthly Tmin (A), Tmax (B), Humidity (C), and precipitation (D), all lagged 1 and 2 months.** The x axis represents increasing variations in the meteorological covariates. The y axis indicates the contribution of the smoother to the fitted values. The y axis is labelled s(cov, edf), where cov indicates the name of the covariate, and edf represents the estimated degrees of freedom of the smooth function used to represent its relationship with number of dengue cases. The red lines indicate the maximum likelihood estimates, and the grey shaded areas represent the 95% confidence intervals. The rug at the bottom of the figures indicate observed values of the covariates.

**Figure 2:**
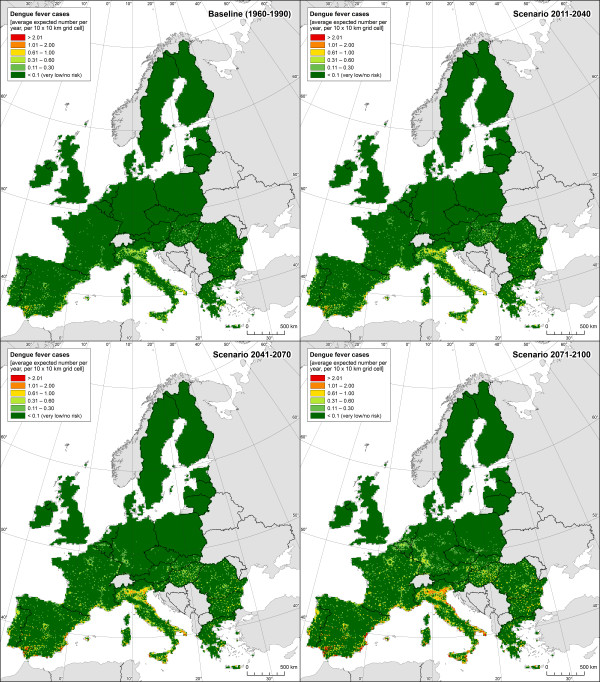
**Average expected number of dengue cases in Europe modelled using GAM model for baseline conditions and climate change scenarios for early, medium and late century.** Number of cases was calculated for each 10 × 10 km grid.

**Figure 3:**
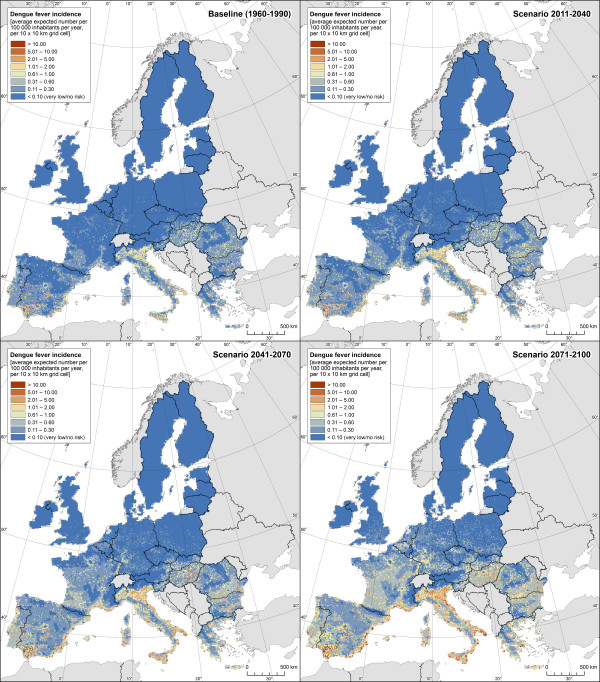
**Dengue fever incidence rate expressed as number of cases per 100 000 inhabitants per year for baseline conditions and climate change scenarios for early, medium and late century.**

**Figure 4:**
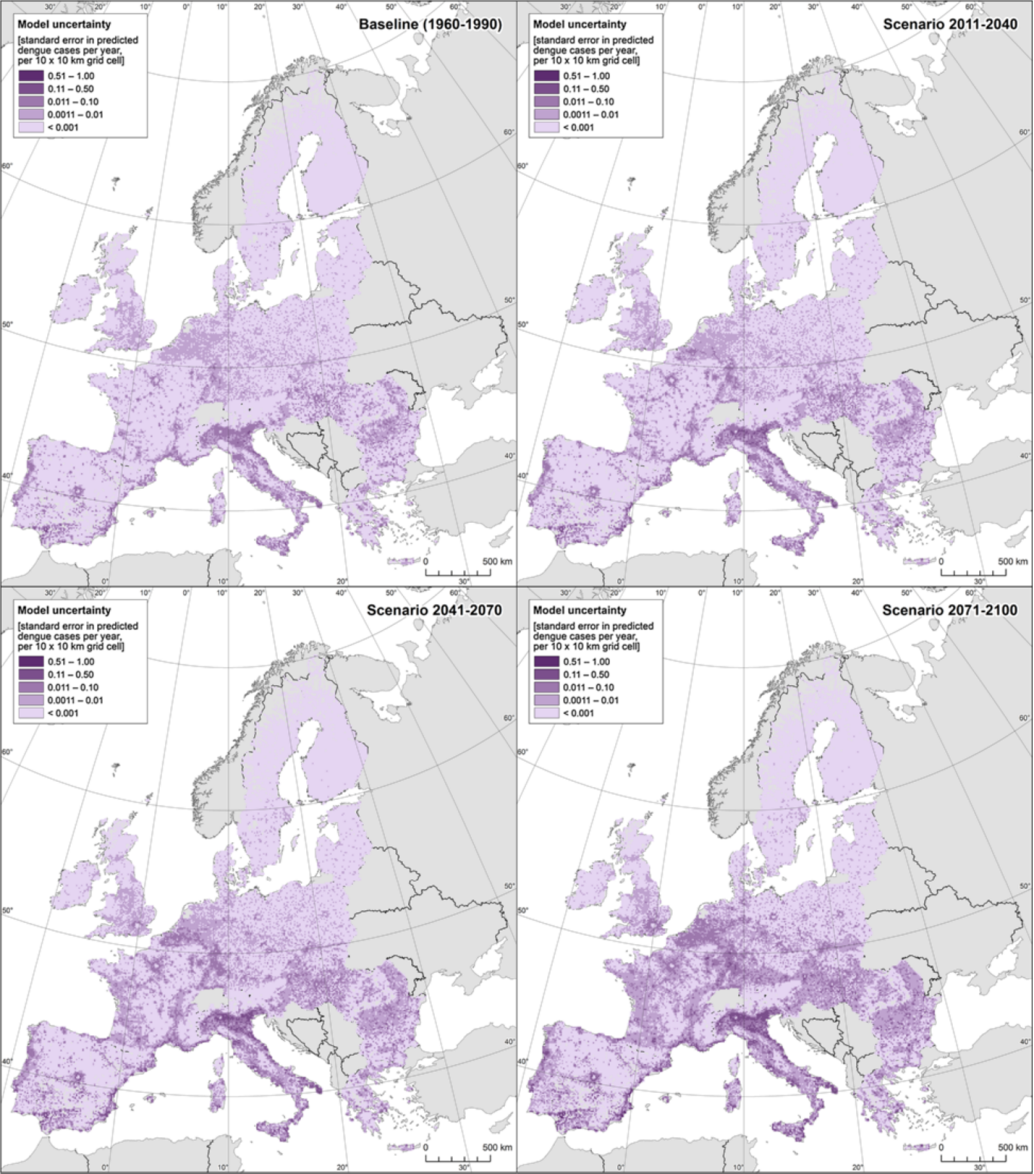
**Maps of uncertainty showing standard errors for projected average number of dengue cases for baseline conditions and climate change scenarios for early, medium and late century.**

The dengue-weather relationships estimated by our Poisson GAM based on the Mexican data are presented in Table 2. The model explained 44% of the deviance within the Mexican dataset. Figure 1 shows the estimated effects of weather variables on number of dengue cases. All climate parameters were statistically significant in a highly non-linear way. The greatest effect was associated with monthly average of minimum temperature followed by monthly relative humidity (both variables lagged one and two months). For socioeconomic variables, population density, degree of urbanization and log population were all significantly associated with dengue incidence (Table 2).The GAM estimated relationships were used to project dengue fever risk in Europe under climate change conditions expressed as dengue cases (cases/year/10×10 km grid). For the baseline period (1960–1990), number of dengue cases are between 0 and 0.6 for most European areas, corresponding to an incidence of less than 2 per 100 000 inhabitants (Figures 2 and 3). Over time, an increase in dengue risk is projected, with highest incidence rates found for the long-term scenario 2070–2100. Indeed, for the baseline period hardly any grid cell had incidence rate exceeding 10 per 100 000 inhabitants, while a substantial amount of grid cells are within this category when considering the long-term-scenario, mostly localised in southern Europe. For each estimated dengue incidence, standard error was calculated and values presented as maps of uncertainty (Figure 4). The general trend was that standard errors tend to correlate with incidence rates, as would be expected from a Poisson model. The maps also highlight that the standard errors are not consistent across the continent.

**Table 2:** **Model estimates of the effects of weather and socioeconomic variables on dengue**

Smooth terms	edf	F
s(Tmin averaged over previous 2 months)	3.95	68.05^†††^
s(Tmax averaged over previous 2 months)	3.28	32.91^†††^
s(Humidity averaged over previous 2 months)	3.94	127.80^†††^
s(Precipitation total over previous 2 months)	2.85	16.90^†††^
**Linear terms**	**Estimate**	**SE**
Intercept	-15.52	0.48
Population density	-0.0028	0.0004^†††^
Urbanisation	0.026	0.002^†††^
GDP	-0.0041	0.007
Log Population	2.54	0.070^†††^
Explained deviance	44.2%	
GCV score	124.8	

^†††^Significant at the 0.0001 level.

edf = effective degrees of freedom of the smooth function terms (edf >1 indicate nonlinear relationships); F value is an approximate F-test, SE = asymptotic standard error. GCV = Generalized Cross Validation.

It can be seen from Figures 2 and 3 that the risk is not equally distributed across Europe. Generally, southern Europe appears at higher risk, with most of the coastal areas being particularly affected. In contrast, northern Europe, the British Isles and most of central Europe show virtually no risk in the baseline period. Over time, the risk in southern Europe and in particular along the Mediterranean coast is projected to increase considerably, with highest incidence revealed along the Italian coast, the Po Valley region, the Spanish Mediterranean coast and southern Spain in general (Figures 2 and 3). In other parts of Europe a change from virtually no risk for the baseline period to incidence rate of up to 10 cases per 100 000 inhabitants are projected, such as in large parts of France, south-western Germany, Hungary, and the Balkan region. By contrast, in northern Europe, for most of the British Isles and the Baltic states, the risk is projected to remain virtually zero even for the long-term scenario 2070–2100.

## Discussion

We have presented the first ever projections of future dengue fever risk in Europe under climate change based on empirical modelling of laboratory confirmed dengue incidence as a function of climate and socioeconomic variables. Our study has shown that the risk of dengue fever is likely to increase in Europe under climate change, but that almost all of the excess risk will fall on the coastal areas of the Mediterranean and Adriatic seas and the North Eastern part of Italy, particularly the Po Valley. Although we have only modelled dengue fever, our findings may have implications for other mosquito-borne diseases such as Chikungunya, which share the same vector species and may have similar transmission patterns to dengue fever.

Previous work in the area has primarily focused on the expected future distribution of the *Aedes* vector [40–44]. Whilst such studies have proven helpful to determine the potential presence of the vector in a given area, the presence of the vector does not necessary translate into disease occurrence. Because our model is based on disease occurrence, the GAM-estimated relationships are likely to be more useful for estimating dengue risk across Europe than models based only on the mere presence of the mosquito. Nevertheless, our results are generally in agreement with the conclusions of projections based on vector distribution. One such example of concordance is Italy, which was identified as a potential dengue hot spot using both approaches (i.e. vector presence and dengue risk). The most noticeable discrepancies for dengue fever risk, however, were associated with Spain and France. In our study, southern and eastern Spain is associated with increased dengue risk, however, *A. albopictus* maps show that this area is likely to be unsuitable by mid/end century [40–43], probably because of hotter and drier weather conditions. On the contrary, France is considered here at medium risk (excluding Mediterranean areas), while it is considered an area of high suitability for *A. albopictus* [40, 41, 43]. Possible explanations for these discrepancies include different climate change scenarios and dissimilar climatic variables incorporated in modelling approaches. A recent study by Rogers and colleagues established a dengue risk map for Europe based on a global risk map taking into account vector presence, disease occurrence and various environmental factors [45]. They showed that while most Europe is at low risk, most major cities combining warmer temperatures and higher population density are highly suitable for dengue transmission. However, as the authors acknowledged, several other factors can influence disease occurrence and transmission. In our study, most cities are not at an increased risk until mid to end of the century. Understandably, it is difficult to compare two risk maps generated using different methodologies and data sets. Nevertheless, European dengue hot spots identified in this and other studies should be made aware of the projected risk.

Herein, we identified a large European geographical area permissive for dengue fever transmission. Whether dengue will become endemic in a particular area depends on various climatic and non-climatic factors, in addition to disease risk in neighbouring areas, which makes estimation of actual incidence problematic. Nevertheless, dengue is unlikely to become endemic in areas of moderate risk, especially if nearby areas have low risk. Consequently we would hypothesise that, should dengue fever become endemic in Europe, it is likely to be primarily in the Mediterranean and Adriatic coastal plains and the Po Valley area of Italy. This does not mean that localised outbreaks of dengue would not occur elsewhere, but that if they did, they would be less likely to be self-sustaining.

For the purpose of this study, we have used Mexican dengue fever surveillance data to project dengue incidence in Europe. This could be considered a limitation because of applicability to European settings and transferability issues. It would clearly have been preferable to use European dengue data but at present there have been too few cases in Europe for any meaningful analysis. In the absence of worthwhile European data, the Mexican dengue surveillance dataset is without doubt the best alternate source of empirical data. The Mexican dataset comprises the largest such set yet assembled with monthly data and sub-national resolution. Furthermore, Mexico is a large country comprising multiple climate zones and thus providing the opportunity of modelling climate impacts on dengue over a wide range of climatic conditions. Mexico is a middle income country, whose socioeconomic status could be comparable to some European countries http://data.worldbank.org/data-catalog/world-development-indicators (last accessed June 2014). Nevertheless, there are several mismatches. One particular issue is seasonal climatic variation, as seasons in Mexico may not match those in Europe. For example, Mexico is highly affected by the El Niño–Southern Oscillation [46], which does not have such an impact in Europe. Similarly, winter climatic conditions can be very different between Mexico and Europe, therefore influencing overwintering and survival of *Aedes* populations. In order to test for this variability, we fitted the model with and without the seasonal term and found that the shape of the estimated relationships graph was similar with a slight difference in order of magnitude. Additional file 2 shows the range of the two weather variables most significantly associated with dengue risk (Tmin and humidity). There is a major area of overlap between the two sufficient to give validity to the use of the Mexico data. The major European areas that do not overlap with Mexico are unlikely to be in areas at risk from dengue. Another important point to consider is the different socioeconomic conditions and cultural habits between Mexico and Europe. Whilst we were not able to model many of these differences due to the absence of adequate data, some of the variables may have little impact on overall dengue risk. For instance, GDP was not found to be significantly associated with dengue fever risk. However, this could be due to data constrains because GDP were available as yearly data that arose from linear interpolations based on 5 years interval. Taking into account these constraints, we have been careful not to be too specific about how many extra cases of dengue we are likely to see in Europe, rather on identifying high risk areas. Clearly even in highly conducive areas, dengue fever will not become endemic if it is not introduced at some point and so the development and spread of dengue endemicity is likely to be a stochastic process, nevertheless it is relying on vector presence, virus introduction and host susceptibility.

An important issue is that the model is produced for a country where dengue is endemic. If dengue was introduced into Europe, then it could spread rapidly in the early years of its establishment and become endemic. This is because almost all Europeans would be immunologically naïve and therefore actual cases could outstrip our projections. A further source of uncertainty would be the adaptation of the European health authorities to the emergence of dengue, including health practitioners’ awareness and effective diagnostic and treatment measures. However, even well-staffed health services with adequate infrastructure could struggle to manage dengue fever [23]. Further response measures that could lower transmission rate, while awaiting the development of an effective vaccine, should include integrated vector management. However, the effectiveness of vector control strategies is not always supported by adequate evidence based evaluations [47]. In addition, unless vector control is performed in a sustainable manner, it is most likely to be inefficient.

Another limitation of the study is related to the mosquito vector. Although, *A. albopictus* is present in Mexico, dengue is mainly transmitted by *A. aegypti.* This primary dengue vector is responsible for major dengue epidemics and the severe life- threatening form of the disease [12]. In Europe, *A. aegypti* is only present in Madeira, where it caused sporadic cases and a sustained dengue outbreak [48]. The main *Aedes* species in Europe is *A. albopictus*. This species is associated with sporadic dengue cases due to its limited competence related to its feeding behaviour and its relative recent adaptation to *flaviviruses* (including dengue fever virus) [49]. Consequently, our results are likely to over-estimate dengue risk in Europe. Additionally, some dengue virus serotypes were shown to cause more severe symptoms and spread more easily [50], therefore the impact of dengue introduction in Europe and subsequent transmission is influenced by virus-vector interaction and the associated risk and severity could either increase or decrease accordingly. Nevertheless, living standards in Europe are likely to limit dengue spread as has been reported in Texas [51], consequently, the actual dengue incidence could be much lower than projected using our model.

One finding that may cause some concern is that under baseline conditions some areas are identified as being at increased risk of dengue fever, when dengue in Europe is effectively non-existent. This is legitimate because the model is projecting areas where, given the provided meteorological and socioeconomic conditions, dengue fever may occur, independently of other confounding factors including vector presence, virus circulation and control measures. Undoubtedly, some European areas are permissive for dengue fever transmission as supported by endogenous cases in France and Croatia in 2010 [8, 9]. The French cases were recorded in Nice, which was indeed highlighted as high risk area in our model. Croatian cases were on the Peljesac peninsula and the island of Korcula (outside the EU27 and therefore not modelled here). Furthermore, in 2007, there was an outbreak of Chikungunya (another viral disease spread by *A. albopictus* mosquitoes) that affected north eastern Italy on the Adriatric coast [52, 53]. Although Chikungunya virus shows adaptive mutation for *A. albopictus* [49], not observed for dengue virus, this area is clearly permissive for the mosquito vector and is associated with the highest projected dengue fever risk in our model. These sporadic cases and outbreaks confirm the general spatial pattern of dengue risk as estimated by our model.

An issue valid for both Mexico and Europe is that Mexican dengue fever data is based on laboratory confirmed reported cases of infection. While on one hand, and in particular for Europe, our approach of modelling dengue risk based on reported cases is considered novel and unique, on the other hand such reports are known to substantially underestimate the actual number of cases because a significant proportion of infections are not diagnosed and reported. A limitation of dengue surveillance in Mexico is its reliance on a passive surveillance system based on unspecific symptoms, coupled with low awareness of health practitioners and limited access to reliable diagnostic tests. Estimation of the sensitivity of dengue surveillance systems varies inter and intra countries but it has been reported that for every recorded case, there may be somewhere between 10–27 cases that go unreported [54]. This could mean that the number of dengue fever cases could be substantially higher than estimated here. It is not known what the sensitivity of dengue surveillance systems in Europe would be, however, asymptomatic and mild cases could go undiagnosed.

In order to assess the impact of climate change on dengue risk in Europe, we used predictions based on the A1B scenario because it is considered more realistic in light of the current global emissions. This is particularly relevant when compared to other more extreme scenarios (such as the low emission SRES B1, or the newer RCP2.6 scenarios) that assume drastic CO_2_ reductions globally in the coming decades, which is unlikely to happen. Running the current model using additional climate change scenarios would add value to the predictions and allow comparative analysis and could be done as a subsequent study. In order to assess the effect of climate change on dengue risk, all non-climatic variables were assumed to remain at their baseline levels, while this could be considered a limitation, some socioeconomic variables were not significantly associated with dengue risk. In addition, population and urbanisation projections for Europe show that minor changes are expected (with some local variation), especially when compared to other parts of the world (where significant increase in population size and urbanisation are expected until 2100) (http://esa.un.org/wpp/ and http://esa.un.org/unpd/wup/ last accessed August 2014).

A recent systematic review of quantitative models assessing the impact of climate change on dengue transmission by Naish and colleagues [55] found that despite using different methodologies, most models consider that temperature is the most important climatic factor driving dengue transmission but that precipitation and humidity are also important, which is in accordance with our model. Despite some methodological issues, most models report increased climatic suitability and expansion of geographical range under various climate change scenarios and in different regions of the world [55]. Improved climate change scenarios and better understanding of vector-borne diseases biology and transmission are likely to contribute to more accurate disease risk models in the future.

## Conclusions

This study allowed modelling of dengue fever risk in Europe based on actual clinical data. The model calibrated under Mexican conditions resulted in reliable and geographically meaningful patterns of projected dengue fever risk in Europe. The risk maps indicate that climate change is likely to contribute to increased dengue risk (and possibly other mosquito-borne diseases) in many parts of Europe, especially towards the end of the century. The areas of greatest increased risk are projected to be clustered around the Mediterranean and Adriatic coasts and in northern Italy. The exact incidence is dependent on several other factors, some of which we were unable to model at this stage (such as vaccine development). Nevertheless public health agencies in high risk areas need to plan, implement and evaluate active entomological reporting and sentinel clinical surveillance and should aim to improve awareness of the increased risk amongst health practitioners and the general public.

## Electronic supplementary material

Additional file 1: **Maps of Gross domestic product (GDP) of 2006 at NUTS-3 level calculated from Word Bank and EUROSTAT data (Left), and degree of urbanisation as derived from the EFGS GEOSTAT 2006 population grid dataset (right).** (PNG 2 MB)

Additional file 2: **Range of Tmin and humidity, the two weather variables most significantly associated with dengue risk, in Mexico and Europe.** (PNG 77 KB)
