# Supplementary figures and images for: Climate change and the emergence of vector-borne diseases in Europe: case study of dengue fever
Source: BMC Public Health. 2014 Aug 22;14:781. doi: 10.1186/1471-2458-14-781 (PMC4143568; doi:10.1186/1471-2458-14-781)

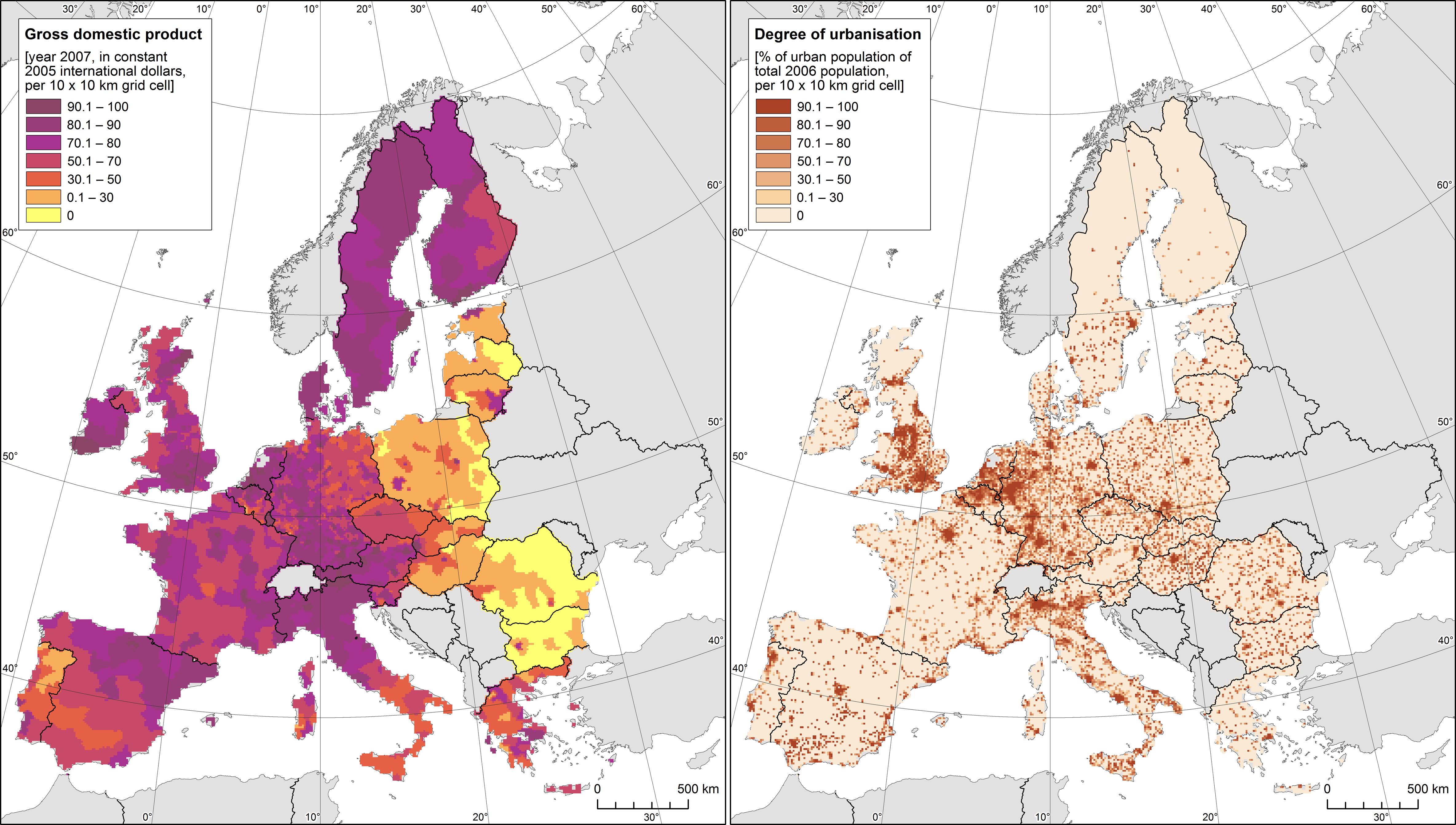

Supplement: Supplementary file 1 — Additional file 1: Maps of Gross domestic product (GDP) of 2006 at NUTS-3 level calculated from Word Bank and EUROSTAT data (Left), and degree of urbanisation as derived from the EFGS GEOSTAT 2006 population grid dataset (right). (PNG 2 MB) [file 12889_2014_6968_MOESM1_ESM.png]

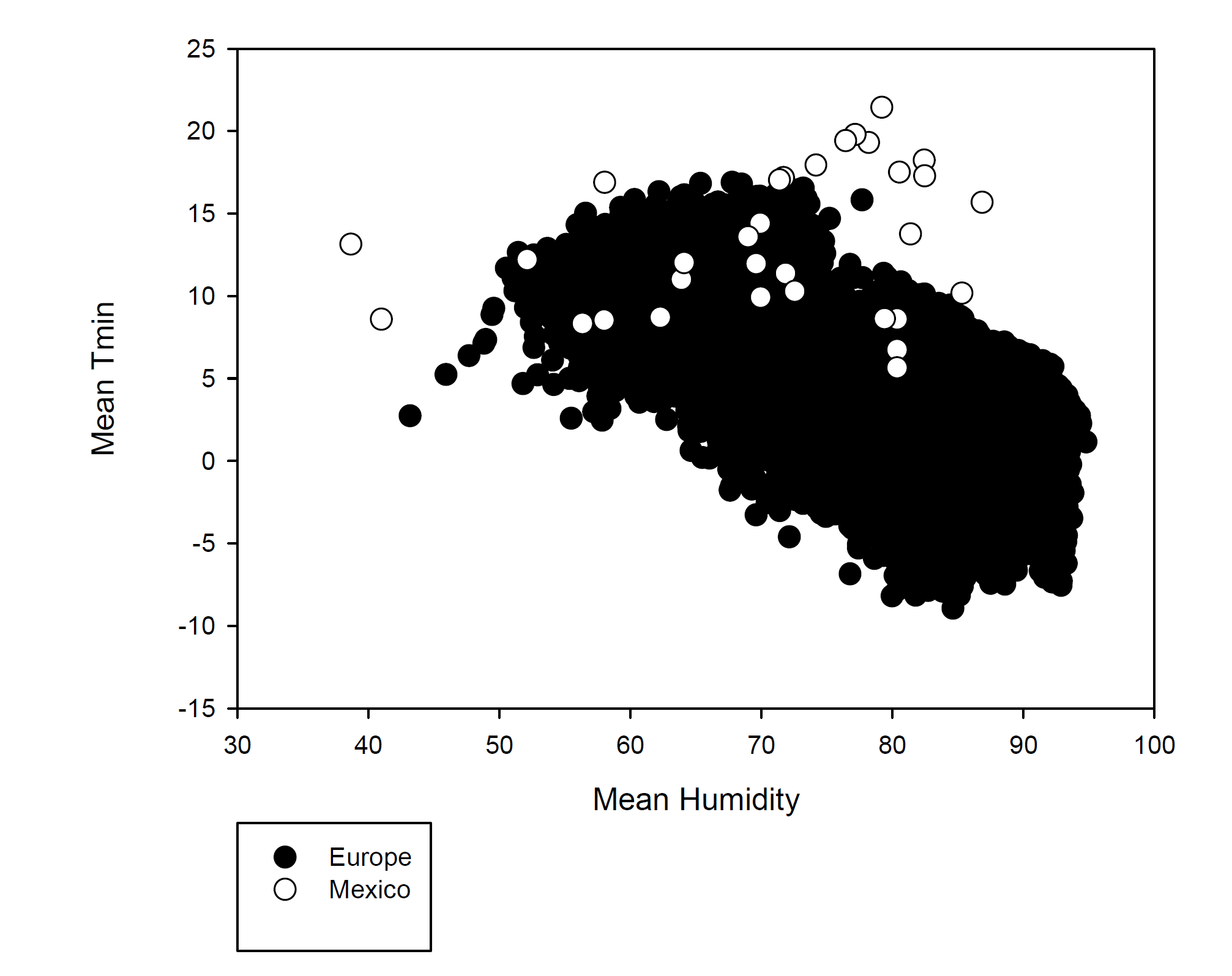

Supplement: Supplementary file 2 — Additional file 2: Range of Tmin and humidity, the two weather variables most significantly associated with dengue risk, in Mexico and Europe. (PNG 77 KB) [file 12889_2014_6968_MOESM2_ESM.png]
